
JOURNAL INFORMATION
==============================
NLM Title Abbreviation: ScientificWorldJournal
Journal ID: ScientificWorldJournal
NLM Journal ID: 101131163
Journal ID: TSWJ

The Scientific World Journal

ISSN: 2356-6140
EISSN: 1537-744X
Publisher: Wiley

ARTICLE INFORMATION
==============================
PMCID: PMC6084010
PMID: 30147541
DOI: 10.1100/tsw.2001.107
Article ID: 243861
Article version: 1
Subjects: Short Report

Microglia and Astrocytes in Neuro-Aids, Alzheimers Disease, and Multiple Sclerosis

Minagar Alireza 1 *
Shapshak Paul 1 2 3
Heyes Melvin 4
Sheremata William A. 1
Fujimara Robert 2
Ownby Ray 2
Goodkin Karl 2
Eisdorfer Karl 2
1 Department of Neurology, University of Miami School of Medicine, Miami, Florida 1501 NW 9th Avenue, Miami, FL 33131, USA
2 Department of Psychiatry and Behavioral Sciences, University of Miami School of Medicine, Miami, Florida 1501 NW 9th Avenue, Miami, FL 33131, USA
3 Department of Pathology, University of Miami School of Medicine, Miami, Florida 1501 NW 9th Avenue, Miami, FL 33131, USA
4 NIMH4, NIH, Bethesda, MD 20014, USA
*Alireza Minagar: aminagar@aol.com
Publication date: 2001
Electronic publication date: 2001 Jul 17
Volume: 1
First page: 69
Last page: 69
Copyright: Copyright © 2001 Alireza Minagar et al.
Copyright year: 2001
License: This is an open access article distributed under the Creative Commons Attribution License, which permits unrestricted use, distribution, and reproduction in any medium, provided the original work is properly cited.
License URL: https://creativecommons.org/licenses/by/3.0/

==============================
Miami *Nature Biotechnology* Short Reports
*TheScientificWorld* (2001) 1(S3), 69SR
ISSN 1532–2246; DOI 10.1100/TSW.2001.107

### MICROGLIA AND ASTROCYTES IN NEURO-AIDS, ALZHEIMER'S DISEASE, AND MULTIPLE SCLEROSIS

Alireza Minagar*[1], Paul Shapshak[1,2,3], Melvin Heyes[4], William A. Sheremata[1], Robert Fujimara[2], Ray Ownby[2], Karl Goodkin[2], and Karl Eisdorfer[2]

Departments of Neurology[1] and Psychiatry and Behavioral Sciences[2], Pathology[3]
University of Miami School of Medicine, Miami, Florida
1501 NW 9th Avenue, Miami, FL 33131
NIMH[4], NIH, Bethesda, MD 20014
* Aminagar@AOL.com

**INTRODUCTION.** The central nervous system (CNS) has traditionally been regarded as an immunologically privileged and isolated organ. This immunoisolation is achieved through the specific structure of the blood brain barrier and CNS immunosuppressive microenvironment.[1,2] However, activated macrophage/microglia (MØ) and astrocytes participate in the pathogenesis of various neurological diseases through expressing of MHC and adhesion molecules and releasing reactive oxygen intermediates, quinolinic acid, chemokines, cytokines, and other components of inflammation. The role of macroglia/macrophages and astrocytes in cellular/molecular mechanisms of pathogenesis of three common dementias; HIV-associated dementia (HAD), Alzheimer's disease (AD), and multiple sclerosis (MS) have been studied. Each disease had its specific clinical course and symptomatology that are well known. At the cellular and molecular levels there are both common and distinct features that are under study and require further elucidation. Recently Link et al.[3] indicated support for roles of both microglia and astrocytes in regulating demyelination in MS. We have generalized this significant concept of neuropathogenesis to HAD and AD. HIV-1 infection is the original insult in Neuro-AIDS. In fact, our results indicate that there are neurovirulent strains of HIV-1 that invade the brain. Furthermore, we have published preliminary studies showing that there is brain regional heterogeneity of macrophage/microglia, virus load, and virus strains. At the cellular level, there is a greater virus load in central nervous system (CNS) macrophage/microglia than in peripheral nervous system (PNS) macrophages. In both tissues, macrophages appear to be involved in pathogenesis and produce toxic molecules including cytokines, chemokines, and nitric oxide (NO). Furthermore, we have demonstrated increased NO synthase in brain interneurons of drug abusers with AIDS associated with increased neuronal death (manuscript under review). The etiologies of AD and MS remain unknown. However, cellular/molecular mechanisms of pathogenesis are specific manifestations of these diseases. For example, it is clear that AD results from production of aberrant eta-amyloid proteins and oligopeptides. Microglia and astrocytes are activated as a result and are associated with further damage. Demyelination in MS involves lipid-laden macrophages perhaps spurred by viral and other antigens. In all three diseases cytokine/chemokine communication between microglia and astrocytes occur and apoptosis is a mechanism of neuronal death. Also, apoptosis of oligodendrocytes occurs in MS.

**METHODS.** Our methods of analysis include quantitative polymerase chain reaction (PCR), and combined *in situ* hybridization and immunohistochemistry. Epidemiological and ethnographic studies as well as subject clinical evaluation were done. In addition, we have extensively reviewed the literature and will report our synthesis of the review.

**RESULTS.** MØ play destructive roles in HAD, AD, and MS while astrocytes play a protective role only in MS. Astrocytes have a destructive role in HAD and AD.

**DISCUSSION.** The goal of this short report is to assess the data relevant to the balance between microglia and astrocytes stimulation and their role in the interactions between brain and the immune system. Activated macrophage/microglia (MØ) and astrocytes participate in pathogenesis of these three neurological diseases through expressing of MHC and adhesion molecules and releasing reactive oxygen intermediates, quinolinic acid, chemokines, cytokines, and other components of inflammation. There is a balance between the MØ and astrocytes and their effects on Th1/Th2 in the brain.[3,4] The players are the same in the three diseases, yet the primary causes of each disease are different enough to result in different courses of pathogenesis. We will diagram and specify the protective as well as pathogenic roles of MØ and astrocytes in the three diseases.

**REFERENCES.**

1. Benveniste, E.N. (1992) Cytokines: influence on glial cell gene expression and function. Chem. Immunol. 52, 106-153
2. Frohman, E.M., Van Den Noort, S., and Gupta, S. (1989) Astrocytes and intracerebral responses. J. Clin. Immunol. 9, 1-9
3. Xiao, G.B. and Link, H. (1999) Trends Immunol. 20, 477-479
4. Xiao, B.-G. and Link, H. (1998) Immune regulation within the central nervous system. J. Neurol. Sci. 157, 1-12
